# Heavy Metal Lead Exposure, Osteoporotic-like Phenotype in an Animal Model, and Depression of Wnt Signaling

**DOI:** 10.1289/ehp.1205374

**Published:** 2012-10-19

**Authors:** Eric E. Beier, Jason R. Maher, Tzong-Jen Sheu, Deborah A. Cory-Slechta, Andrew J. Berger, Michael J. Zuscik, J. Edward Puzas

**Affiliations:** 1 Center for Musculoskeletal Research, and; 2 Department of Environmental Medicine, University of Rochester, School of Medicine and Dentistry, Rochester, New York, USA; 3 The Institute of Optics, University of Rochester, Rochester, New York, USA

**Keywords:** bone mineral density, lead, mesenchymal stem cells, rat, Wnt signaling

## Abstract

Background: Exposure to lead (Pb) from environmental and industrial sources remains an overlooked serious public health risk. Elucidating the effect of Pb on bone cell function is therefore critical for understanding its risk associated with diseases of low bone mass.

Objectives: We tested the hypothesis that Pb negatively affects bone mass. We also assessed the underlying mechanisms of Pb on bone signaling pathways.

Methods: We used a model of low-level Pb exposure in a rodent beginning before conception and continuing over 18 months. We characterized the effect of Pb on bone quality using dual-energy X-ray absorptiometry (DXA), micro-computed tomography, Raman spectroscopy, and histology. We assessed the effect of Pb on bone and adipocyte formation by mineral deposition, lipid droplet formation, and Western blot and RNA analysis.

Results: Pb-exposed animals had decreased bone mass that resulted in bones that were more susceptible to fracture. Pb decreased osteoblastic cell number leading to a depression of bone formation. Accompanying this, Pb exposure elevated sclerostin protein levels in the skeleton, and correspondingly reduced levels of β-catenin and Runx2 in stromal precursor cells. Pb also increased skeletal expression of peroxisome proliferator-activated receptor-γ (*PPAR-*γ). These results indicate a shift in mesenchymal differentiation wherein Pb promoted enhanced adipogenesis and decreased osteoblastogenesis. Substantial differences in bone marrow composition were observed, highlighted by an increase in adipocytes.

Conclusions: The disruption Pb has on bone mass and bone homeostasis is principally explained by inhibition of the Wnt/β-catenin pathway, which may provide a molecular basis for novel therapeutic strategies to combat Pb-induced bone pathologies.

Although attempts have been made to decrease the amount of lead (Pb) in the environment, it remains a pervasive toxicant contributing to problems for human health. In the 1970s, > 75% of Americans had blood Pb levels (BLL) > 10 µg/dL (Mahaffey et al. 1982), which is where the current adopted threshold of concern resides as defined by the Centers for Disease Control and Prevention (1991). Although the average BLL has declined, developmental and lifelong low Pb exposures are recognized as having a persistent negative impact on human health (Goyer 1993). These serious Pb-related health issues, notably in the skeleton, can occur at levels below the current threshold BLL of concern.

Although effects of Pb have previously been underappreciated, recent clinical and basic science research has suggested that Pb has a profound influence on both the developing and adult skeleton. Reports in animals (Bagchi and Preuss 2005; Escribano et al. 1997; Gruber et al. 1997) and humans (Campbell and Auinger 2007; Nash et al. 2004) have begun to associate a detrimental impact of cumulative Pb burden on bone mineral density (BMD) and development of osteopenia. In a study of rats exposed to increasing levels of Pb in their drinking water for 6 weeks, Bagchi and Preuss (2005) reported an inverse correlation between Pb exposure and decreased total body BMD. In the Third National Health and Nutrition Examination Survey (NHANES III, 1988–1994), Nash et al. (2004) also found a significant inverse correlation between BLLs and femoral BMD. Despite these observations, attempts at describing a mechanism of skeletal Pb toxicity remain elusive.

Osteoporosis is a progressive disease characterized by a reduction in BMD sufficient to reduce biomechanical strength and increase fracture risk. We postulate that decreased osteoblast activity induced by Pb subsequently weakens skeletal structure and thus increases the risk of osteoporosis. This is supported uniformly by numerous reports that Pb negatively affects osteoblast function *in vitro* (Dowd et al. 2001; Klein and Wiren 1993; Puzas et al. 2004; Sauk et al. 1992).

One key signaling pathway coordinating bone homeostasis is the Wnt/β-catenin pathway. Activation of this pathway occurs with binding of Wnt agonists to the frizzled receptor, which stabilizes β-catenin (unphosphorylated) in the cytoplasm as a result of a disassociation of the negatively regulating complex consisting of glycogen synthase kinase 3β, Axin, Frat1, and Disheveled. There have been numerous efforts in recent years to delineate this pathway and understand the impact of bone-specific Wnt molecules. Several genetic models have illustrated the importance of β-catenin’s function on mesenchymal lineage specification (Shahnazari et al. 2008) and on bone homeostasis (Johnson et al. 2004; Robinson et al. 2006). These efforts led to the discovery of the Wnt antagonist, sclerostin (Johnson et al. 1997), which was shown to bind to low-density lipoprotein receptor-related protein 5 (LRP5) and LRP6 with high affinity co-receptors that are required for transduction of canonical Wnt signals (Li et al. 2005). Sclerostin was further documented to be a potent inhibitor of bone formation through its repression of osteoblast function (Li et al. 2008, 2009; van Bezooijen 2005). Studies from our laboratory have indicated a strong induction of sclerostin by Pb that could provide new insights into a molecular mechanism of disruption of bone homeostasis by inhibition of Wnt signaling.

Our objective in this study was to evaluate bone quality in female rats exposed to physiologically low levels of Pb over a lifetime, mimicking a plausible human exposure. Here we describe the observed phenotype, with specific focus on clinical correlates of dual-energy X-ray absorptiometry (DXA) analysis, chemical composition using optical spectroscopy, and biomechanical strength testing. We also explored the modulation of Pb on Wnt signaling as the driving force for these effects.

## Materials and Methods

*Animals and Pb exposure.* We randomly split Long-Evans rats into two groups. To obtain elevated Pb body burden at conception, we provided rats with water containing either 0 or 50 ppm Pb acetate 2 months before breeding. Female offspring (*n* = 9/group) were continuously exposed to Pb for their lifetimes (Cory-Slechta et al. 2010). Blood Pb samples collected by tail nicks were analyzed by anodic stripping voltammetry using the Lead Care II system (Magellan Diagnostics, Billerica, MA). We then anesthetized the animals and perfused them with either phosphate buffered saline (PBS) or 10% formalin. We harvested skeletal elements and assessed phenotype. All animals were treated humanely and with regard for alleviation of suffering.

*Bone Pb determination, RNA isolation and real-time reverse transcription-polymerase chain reaction (RT-PCR).* We isolated right tibiae, discarded the epiphyses and soft tissues, and flushed the bone marrow with a 25-gauge 5/8-in. needle (Becton-Dickinson, Franklin Lakes, NJ). We homogenized four bones per treatment group and extracted RNA using TRI Reagent (MRC Inc., Cincinnati, OH) according to the manufacturer’s recommendation; this was followed by DNase digestion and column separation using QIAGEN mini columns (QIAGEN, Valencia, CA). We then performed reverse transcription using the iScript cDNA synthesis kit from Bio-Rad (Hercules, CA), and carried out RT-PCR reactions using PerfeCTa SYBER green (Quanta BioSciences Inc., Gaithersburg, MD) according to the manufacturer’s protocols. The gene of interest was normalized with β-actin expression. Primer sequences are available in Supplemental Material, Table S1 (http://dx.doi.org/10.1289/ehp.1205374). For determination of bone Pb, we washed four flushed diaphyseal bones per group with PBS and incubated them in 3% hydrogen peroxide for 20 min. We then processed the bones as described previously (Carmouche et al. 2005; Parsons 1993) and analyzed the samples using atomic absorption spectroscopy.

*Radiography, bone densitometry, and micro-CT (micro-computed tomography).* We obtained radiographic images using a Faxitron cabinet X-ray system (Faxitron, Wheeling, IL) on nine rats per treatment group. The images were thresholded to remove soft tissue elements. We determined areal BMD from rats *ex vivo* by DXA (Lunar Prodigy Advance; GE Healthcare, Madison, WI). The region of interest (ROI) included the lumbar vertebrae (LV_1_–LV_5_) and femur/tibia.

We determined bone properties using a 10.5-µm resolution VivaCT40 micro-CT scanner (Scanco Medical, Basserdorf, Switzerland), as described previously (Guo et al. 2008). For analyses of trabecular bone within the distal femur and proximal tibia, a region equivalent to 8% of the femur height (2.6 mm) was selected beginning 0.3 mm from the most proximal aspect of the growth plate scanned; images were reconstructed to an isotropic voxel size of 15 μm. We segmented trabecular bone from the cortex using a semiautomated contouring algorithm in the axial plane. We selected LV_3_ for spine, and the ROI included the middle one-third of the vertebral bodies, equivalent to 2.1 mm. For cortical analysis, we chose a region of 0.75 mm along the femoral midshaft.

*Mechanical testing.* We prepared six specimens per group for strength testing of the fourth lumbar vertebral bodies using a low-speed diamond saw to remove posterior elements and endplates. We performed destructive four-point bend tests of the right femurs with a span between the two lower supports set at 14 mm, and the span between the two upper loads set at 7 mm (*n* = 9 rats/group). We conducted testing at a displacement rate of 5 mm/min. Structural parameters, maximum load, stiffness, and energy absorption data were generated from the load-displacement curve for each specimen (Instron 4465/5500; Instron, Norwood, MA).

*Raman spectroscopy analysis.* We assessed cortical bone mineralization by calculating the intensity of several vibrational bonds, which are highly specific to chemical content, using the Raman scattering effect. We chose four femurs from each group based on disparities in femoral bone volume. We acquired spectra from the bone surface on the anterior side of the proximal, distal, and mid-diaphysis regions with an exposure time of 300 sec per region. The locally constructed Raman spectroscopy system used to acquire these spectra has been described previously (Maher et al. 2011). This instrument has a large depth of focus (the illumination numerical aperture is 0.05). Therefore, each measurement probed a large, macroscopic volume of cortical bone. We characterized differences in mineral and protein content between the rat femurs by metrics related to bone biochemistry, including the mineral to matrix ratio [MTMR; peak area ratio of phosphate to methylene (PO_4_^3–^/CH_2_)]. For additional details, see Supplemental Material, p. 3 (http://dx.doi.org/10.1289/ehp.1205374)]. We did not explore the effects of polarization in this study because the illumination was not highly polarized.

*Bone histomorphometry and immunohistochemistry.* Proximal tibia stripped of soft tissues were fixed in formalin for 4 days, decalcified, embedded in paraffin, and sectioned as described previously (Carmouche et al. 2005). Static parameters were calculated using Osteomeasure bone analysis software (Osteometrics, Decatur, GA). The ROI for tibial trabecular bone was an area (1.23 mm^2^) below the growth plate within the metaphysis. For intramedullary fat analysis, we counted the number of fat vacuoles in bone marrow, which appear optically empty in sections. For immunohistochemistry, we deparaffinized sections from three rats per group in xylene; antigen retrieval was performed in 10 mM citrate buffer, pH 6.0, for 1 hr at 80°C for β-catenin (1:30, Cell Signaling Technologies, Danvers, MA) and for 30 min for both sclerostin (Scl; 1:50; R&D Systems, Minneapolis, MN) and runt-related transcription factor 2 (Runx2; 1:100; MLB International, Woburn, MA). Sections were incubated with primary antibodies overnight at 4°C, washed in PBS, and incubated in the appropriate secondary antibodies (1:200; Vector Laboratories, Burlingame, CA) for 30 min. Sections were then incubated with horseradish peroxide streptavidin (1:750; Zymed, San Francisco, CA) for 30 min and developed with AEC (3-amino-9-ethylcarbazole) chromogen for 10 min.

*Cellular assays.* Osteoblastogenesis. We isolated primary rat calvarial osteoblasts from 3-day-old pups as described previously (Ryan et al. 2007). Cells were cultured in osteogenic alpha-MEM (alpha-minimum essential medium) containing Pb at doses ranging from 0.8 µM to 5.0 µM. After 18 days in culture, the cells were washed twice with normal saline, fixed with 10% formalin, and stained with 0.1% alizarin red (95% ethanol) for 20 min.

Adipogenesis. During the proliferative phase and early confluence, C3H10t1/2 mouse embryonic mesenchymal cells were pretreated for 5 days with Pb in high-glucose Dulbecco’s modiﬁed Eagle’s medium (DMEM) with 10% FBS and 1% penicillin/streptomycin. Two days postconfluence, the cells were treated with a hormone cocktail of methylisobutylxanthine, dexamethasone, and insulin in Pb-free complete DMEM, in accordance with standard adipogenesis protocols in this cell line (Cho and Jefcoate 2004). Five days later, we stained lipid droplets with Oil Red O [2.1 mg/mL (4 isopropanol:3 water)] for 1 hr at room temperature. We achieved quantification of the stained area by dissolving stain in 4% IGEPAL (vol/vol isopropanol) and measuring absorption at 490 nm.

We isolated total RNA and protein on day 10 of Pb treatment for osteoblasts and on day 5 for adipocytes; Pb treatment ranged from 0.8 µM to 5.0 µM for RNA and from 1.0 µM to 5.0 µM for protein. We used the RNeasy Plus Mini Kit (QIAGEN) for RNA isolation according to the manufacturer’s protocols. RT-PCR reactions were performed as described above. We conducted protein analysis as described previously by Ryan et al. (2007) using the following antibodies: polyclonal rabbit Runx2, collagen 1 (Col1), or β-actin, all from Abcam (Cambridge, MA); peroxisome proliferator-activated receptor-γ (PPARγ), CCAAT/enhancer-binding proteins β (C/EBPβ) or α (C/EBPα), all from Santa Cruz Biotechnology (Santa Cruz, CA); monoclonal mouse β-catenin (Santa Cruz Biotechnology); or polyclonal goat Scl and Dickkopf1 (DKK1), both from R&D Systems.

*Statistical analysis.* Results are expressed as the sample mean ± SE. We used an unpaired Student’s *t*-test to determine statistical differences, with *p* ≤ 0.05; we used one-way analysis of variance followed by the Tukey multiple comparison test to determine dose-dependent effects. In addition, we used univariate regression to predict the energy to failure of each femur based on the Raman MTMR. We used a leave-one-out cross-validation approach, and prediction accuracy was quantified by calculating the squared sample correlation coefficient (*r*^2^).

## Results

**Figure 1:**
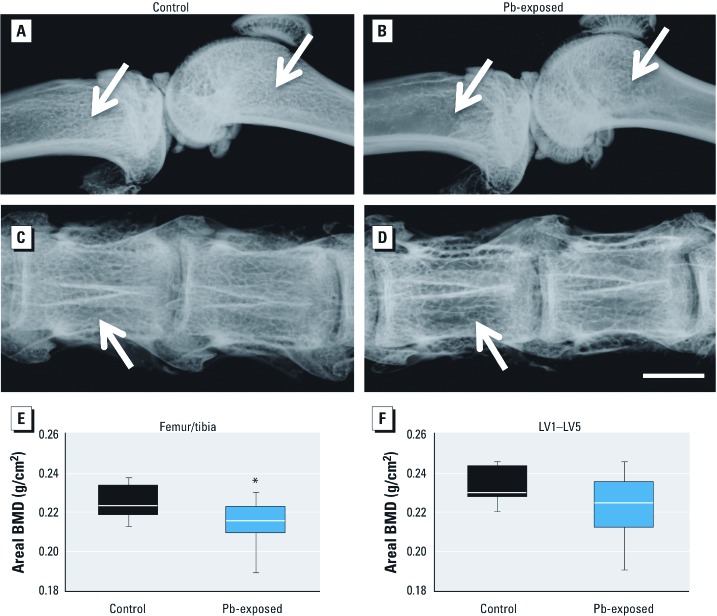
Pb exposure decreased bone mineral density in long bones and lumbar vertebrae of rats continuously exposed to 50 ppm Pb in drinking water. Radiographic images of femur/tibia (*A,B*) and vertebrae (*C,D*) taken *ex vivo* from randomly selected rats are representative of each treatment group. Radiolucency in the trabecular region (arrows) is prominent in Pb-exposed rats (*B*,*D*) but is absent in control animals (*A*,*C*). Bar = 2.5 mm. DXA scans detected lower areal BMD in both leg (*E*) and spine (*F*) of Pb-exposed rats. Data represent mean ± SE (*n* = 9 rats/group). *p < 0.05.

*Effect of Pb on tissue exposure and BMD in aged rats.* During the course of treatment, we monitored Pb exposure levels in the rats (Cory-Slechta et al. 2010). In 18-month-old rats, the background BLL was 0.19 µg/dL in unexposed animals compared with 9.16 µg/dL in Pb-exposed animals. The Pb content in tibia was 30.99 μg/g dry bone, versus 0.17 μg/g in control rats. We took X-rays of whole limbs and lumbar spinal columns *ex vivo* (Figure 1A–D). Radiographs of Pb-exposed animals (Figure 1B,D) show a decrease in trabecular bone in both the long bones and spine, as visualized by increased radiolucency in these regions. *Ex vivo* DXA analysis demonstrated that, compared with control animals, Pb-exposed rats had significantly decreased areal BMD at the femur/tibia region (–4.7%; *p* = 0.04) and a trend toward decreased BMD at LV1–LV5 (–4.9%; *p* = 0.09) (Figure 1E,F).

**Figure 2:**
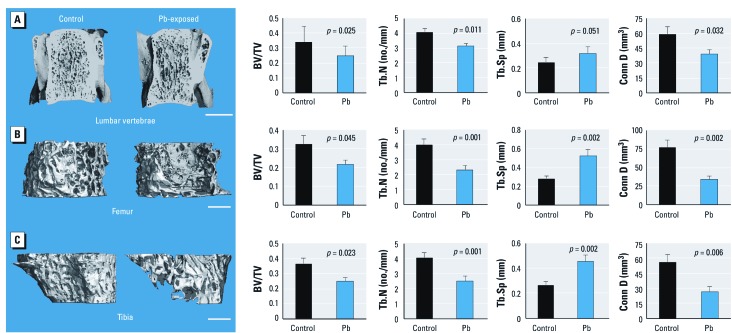
Rats exposed to 50 ppm Pb in drinking water showed a systemic decrease in trabecular bone volume. We analyzed trabecular bone properties in the LV3 (*A*), the distal femur (*B*) and the proximal tibia (*C*). Images (left) are representative transverse sections from control and Pb-exposed rats and were selected based on the median BV/TV; bars = 2.5 mm (*A*) and 1 mm (*B*,*C*). Quantitative analysis (right) shows significant changes in BV/TV, Tb.N, Tb.Sp, and Conn D. Data are mean ± SE (*n* = 9 rats/group).

**Figure 3:**
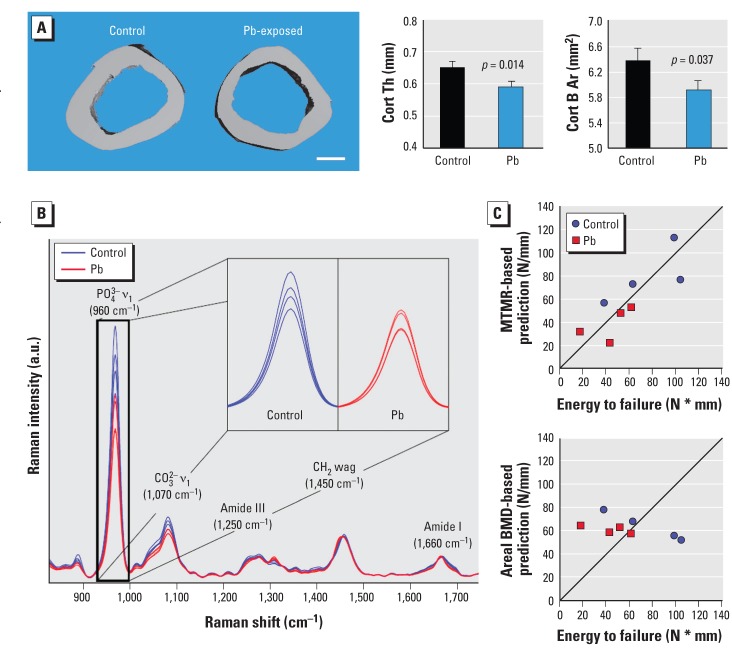
Bone properties at the femoral midshaft were altered in rats exposed to 50 ppm Pb in drinking water. (*A*) Results of micro-CT analysis of rat femurs. (Left) Cross-sectional images representative of treatment group (bar = 1 mm); (right) cortical thickness (Cort Th) and bone area (Cort B Ar). (*B*) Average Raman spectrum acquired from each femur, with the difference in phosphate chemical peaks expanded; Raman intensity is shown in arbitrary units (a.u.). (*C*) Scatter plots show the MTMR‑based (top) and areal BMD‑based (bottom) predictions of the energy to failure for each femur versus its measured value [Newtons per millimeter (N/mm)] [see Supplemental Material, Table S3 (http://dx.doi.org/10.1289/ehp.1205374)]. Data represent mean ± SE for 9 rats/group (*A*) and 4 rats/group (*B,C*).

We performed micro-CT analysis of trabecular bone at the LV3 (Figure 2A). Quantitative measures of trabecular bone quality showed a significant decrease in trabecular bone volume to total bone (BV/TV; –27%), trabecular number (Tb.N; –23%), and connective density (Conn D; –34%), and a significant increase in trabecular bone spacing (Tb.Sp; +30%) in Pb-treated rats over controls. Similarly, the metaphyseal region of the distal femur showed a reduction of trabecular BV/TV after Pb exposure. Quantitative analysis of trabecular bone of the femur showed that Pb-treated rats had significantly depressed bone properties (BV/TV, –33%; Tb.N, –48%; Conn D, –61%; Tb.Sp, +100%) compared with controls (Figure 2B). Micro-CT analysis of trabecular bone in the proximal tibia showed an analogous decrease in BV/TV (–32%) for Pb-treated rats compared with control animals (Figure 2C). Trabecular thickness was unchanged in all bones from Pb-exposed rats; therefore, the decreases in bone volume in Pb-exposed animals could be attributed to the significant reduction in Tb.N (–44%). This corresponded to an increase in Tb.Sp (+83%) and a decrease in Conn D (–61%). All together these data indicate that low-level Pb contributes to a systemic decrease in skeletal mass. Micro-CT analysis of the femoral midshaft showed that Pb-treated rats had significantly decreased cortical bone area (–7%) and cortical thickness (–8%) compared with controls (Figure 3A).

*Biomechanical strength is altered in Pb treatment at the lumbar vertebrae and femoral midshaft.* To assess bone strength, we conducted compression testing of the LV3 and measured maximum load, stiffness, and energy to failure. In vertebrae of Pb-treated rats, we observed reduced vertebral bone strength and significant decreases in maximum load (–35%) and maximum stiffness (–28%) compared with controls [see Supplemental Material, Table S2 (http://dx.doi.org/10.1289/ehp.1205374)]. The decreases in femoral bone mass and thickness observed in micro-CT of Pb-treated rats translated into lower bone strength, as shown by significant decreases in both maximum load (–23%), energy to failure (–31%), and force at yield (–26%) in Pb-treated animals compared with controls

*Effect of Pb on biochemical composition of bone as measured by Raman spectroscopy.* Figure 3B presents the average Raman spectra from rat femurs that have been overlaid; major Raman peaks associated with mineral (PO_4_^3–^ and CO_3_^2–^) and protein matrix [amide III, CH_2_, and amide I] content are indicated. The intensities of several peaks (e.g., PO_4_^3–^ and CO_3_^2–^) were quantified by the Raman parameters. [see Supplemental Material, Table S3 (http://dx.doi.org/10.1289/ehp.1205374)]. The divergence in each of these parameters was statistically significant; the MTMR and crystallinity were smaller and the carbonate to phosphate ratio and collagen maturity were greater in Pb-exposed rats. Scatter plots of energy to failure predicted by univariate regression against the Raman spectroscopy-derived MTMR and the DXA areal BMD are presented in Figure 3C. The MTMR-based predictions were more highly correlated (*r*^2^ = 0.81) with the energy to failure than were predictions based on individual micro-CT parameter (*r*^2^ < 0.6) or areal BMD (*r*^2^ = 0.03).

**Figure 4:**
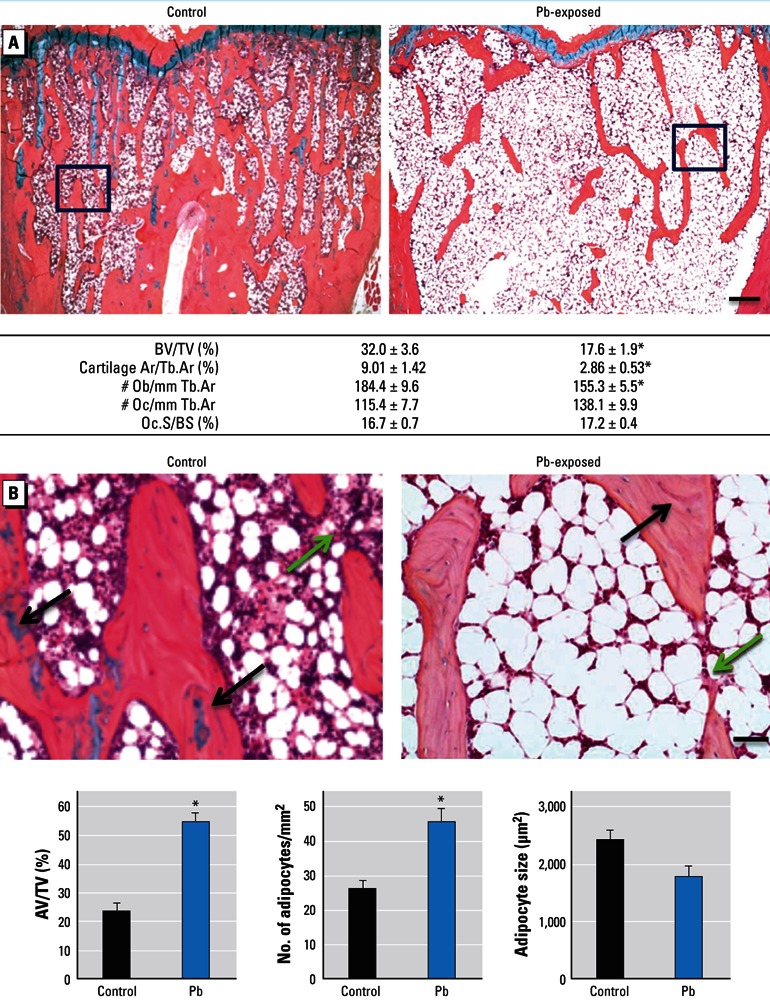
Changes in bone and adipogenic histomorphometric parameters in Pb-exposed rats compared with controls. (*A*) Sections of trabecular bone in the metaphyseal region of proximal tibia from control and Pb-treated rats (top) were evaluated for the following bone properties (bottom): BV/TV, cartilage area to trabecular area (Cartilage Ar/Tb.Ar), osteoblast number to trabecular area (Ob/Tb.Ar), osteoclast number to trabecular area (Oc/Tb.Ar), and osteoclast surface to bone surface (Oc.S/BS). (*B*) Images magnified from areas indicated by black boxes in (*A*) show fatty bone marrow changes in rat tibia (top). Green arrows highlight areas of unfilled tunneling and resorption space in Pb exposures, and black arrows indicate cartilage bars in trabecular bone. Bone sections were evaluated for adipocyte content (bottom). AV/TV, adipose volume to total volume. Data represent mean ± SE (*n* = 4 rats/group). Bar = 100 µm in (*A*) and 500 µm in (*B*). *p < 0.05.

*Histomorphometric analysis of proximal tibia.* To further determine the effect of Pb on bone, we calculated histomorphometric parameters relating to bone formation and bone resorption in trabecular bone of the proximal tibia. In Pb-treated rats compared with controls, we observed a consistent decrease in trabecular bone volume (BV/TV, –56%) (Figure 4A). We also observed significant reductions in the area of cartilage bars present (–70%) and in the number of osteoblasts lining trabeculae. Histology-based bone resorption parameters (osteoclast number to trabecular area ratio, osteoclast surface to bone surface ratio) remained approximately the same in Pb-exposed animals compared with controls. There was no apparent change in the amount or structure of woven bone between the two treatment groups [see Supplemental Material, Figure S1C (http://dx.doi.org/10.1289/ehp.1205374)]. TUNEL staining showed no change in cell viability in the osteocytes in trabecular and cortical bone of Pb-treated rats compared with controls (see Supplemental Material, Figure S1A,B). We determined bone marrow composition by comparing the adipocyte content within the trabecular region. Adipocyte volume (+112%) and number (+52%) were increased, but adipocyte size was not significantly different (Figure 4B).

**Figure 5:**
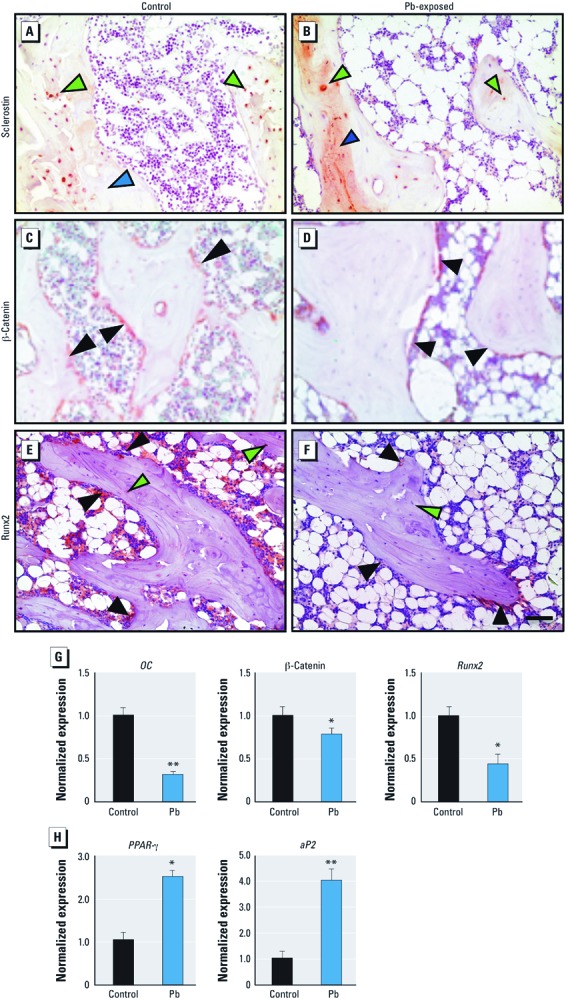
Pb decreases osteogenesis by up-regulation of sclerostin and corresponding suppression of Wnt signaling and osteoblastic genes while also increasing adipogenesis. (*A–F*) Representative immunohistochemical staining for sclerostin (*A,B*), β-catenin (*C,D*), and RunX2 (*E,F*) proteins in proximal tibia of control (*A,C,E*) and Pb-exposed (*B,D,F*) rats. Green arrows indicate osteocytes, black arrows indicate stromal cells, and blue arrows highlight bone matrix (bar = 500 µm). Expression of osteocalcin, β-catenin, and *Runx-2* (*G*), as well as *PPAR-*γ and *aP2* (*H*) mRNA from isolated total RNA in rat tibias using RT-PCR. Data represent mean ± SE (*n* = 4 rats/group). *p < 0.05. **p < 0.005.

*Pb effects on Wnt signaling.* To uncover the molecular signaling changes fundamental to the apparent decrease in bone formation properties and the increase in bone marrow adiposity, we measured the expression of several important proteins essential for these processes. Scl is robustly expressed in mature osteoblasts and osteocytes and is present in the bone matrix (Figure 5A,B). In Pb-exposed rats, we observed an increase in Scl staining around the epiphyseal region in osteocytes in trabecular bone and increased deposition in the bone matrix. Expression of β-catenin was concentrated in the osteoprogenitor cells that line the trabecular regions (Figure 5C,D). In Pb-exposed rats compared with controls, staining for β-catenin showed a decrease in frequency and intensity of expression in cells of the stromal lining. Runx2, a key transcriptional regulator of bone formation, was abundantly expressed in osteoblasts and osteocytes; however, the number of positive cells and the staining intensity were dramatically decreased in Pb-exposed animals (Figure 5E,F).

Real-time PCR analysis of mRNA levels of the bone-forming gene osteocalcin (–68%), the Wnt signaling molecule β-catenin (–26%) and the osteoblast differentiation transcription factor *Runx2* (–57%) were decreased with Pb exposure (Figure 5G), which is consistent with histological findings. At the same time, mRNA levels of proadipogenic genes *PPAR-*γ (+253%) and adipocyte protein 2 (*aP2*) (+389%) were elevated in Pb-exposed animals (Figure 5H).

**Figure 6:**
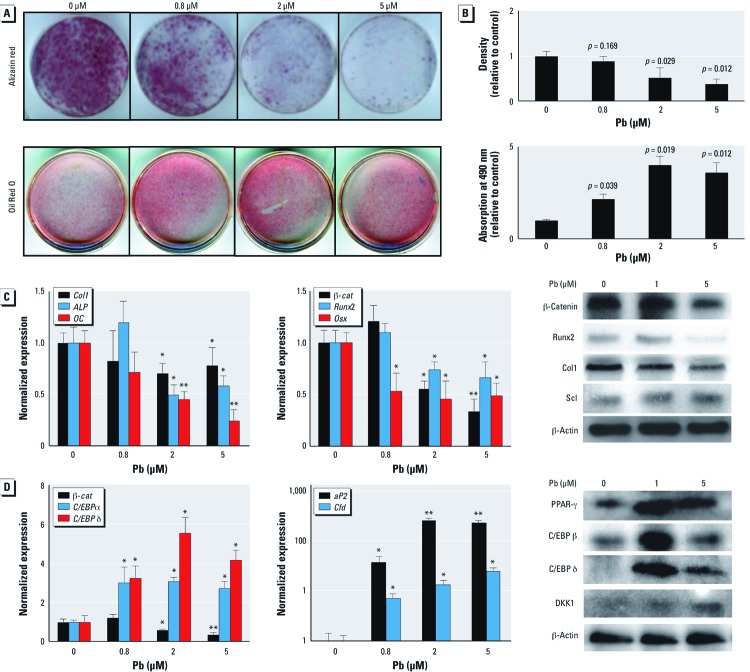
Pb increases the adipogenic potential of C3H10t1/2 mouse embryonic mesenchymal cells and suppresses osteoblast differentiation by corresponding reduction of Wnt signaling. Calvarial osteoblasts and C3H10t1 cells were treated with 0 (control), 0.8, 2, and 5 µM Pb. (*A*) Pb dose-dependently inhibited mineralization of primary osteoblasts, as seen with alizarin red stain (top), and increased the adipogenic potential in C3H10t1/2 cells treated with an adipogenic cocktail and stained with Oil Red O (bottom). (*B*) Quantification of staining shown in (*A*). (*C,D*) Results of RT‑PCR and Western blots showing expression profiles of osteoblastic genes and protein levels after 10 days of Pb exposure (*C*) and expression profiles of adipogenic genes and protein levels after 5 days of Pb exposure (*D*). Data represent mean ± SE for three trials. *p < 0.05 **p < 0.005.

*Pb induces adipogenesis and inhibits bone nodule formation.* We set out to determine the effect of Pb on two aspects of bone homeostasis: mesenchymal cell differentiation to adipocytes and to osteoblasts. Alizarin Red staining revealed that primary cultures treated with Pb (0.8–5 µM) produced significantly less mineralized nodules in a dose-dependent fashion (Figure 6A,B). This indicates a direct inhibitory effect of Pb on osteoblast differentiation *in vitro*. Consistent with this, we observed a reduction in expression of the osteoblastic genes *Col1*, alkaline phosphatase (*ALP*), and osteocalcin (*OC*) (Figure 6C). Expression of pro-osteoblastic transcription factors β*-*catenin, *Runx-2*, and osterix (*Osx*) were also dose-dependently decreased by Pb, and we observed an elevation in Scl protein following Pb exposure.

Alternatively, pretreatment of Pb enhanced the adipogenic potential of C3H10t1/2 cells *in vitro*. Oil Red O staining revealed a 4-fold increase in lipid droplets as a result of exposure to 2 µM Pb (Figure 6A,B). Enhanced adipogenesis was supported by the increased expression of proadipogenic factors *PPAR-*γ, *C/EBP* isoforms, *aP2*, and complement factor D (*Cdf*) (Figure 6D). This was accompanied by an elevation of DKK1.

## Discussion

Our results indicate that long-term Pb exposure in adult rats (BLLs < 10 µg/dL) evokes significant skeletal changes consistent with a low bone mass phenotype resembling osteoporosis. We designed the exposure paradigm used in this study to represent one that is relevant to current environmental exposure in the United States. We used the lowest cumulative exposure studied to evaluate the effect of Pb on bone quality. Bone densitometry measurements demonstrated lower bone mass and a corresponding decreased resistance to biomechanical forces, suggesting a higher susceptibility to fracture. We observed deterioration of trabecular architecture, decreased bone mass, and decreased cortical thickness, all of which have been implicated in reduced bone strength and increased fracture incidence in humans (Barth et al. 1992; Wachter et al. 2002). In addition, the observed biochemical differences in mineral and protein matrix content, as measured by Raman spectroscopy, suggest that long-term Pb exposure causes specific chemical changes in bone, which indicates some degree of altered cell function of osteocytes within the cortical bone sites. These findings are in agreement with a previous report demonstrating reduction in mineral crystal size and decreased MTMR in bones of Pb-exposed mice (Monir et al. 2010).

Decreased BMD and an increased risk of fracture are hallmarks of osteoporosis; thus, our observed association of decreased BMD and bone strength after Pb exposure raises concern for Pb as a risk factor for osteoporosis in aged individuals. Data on human bone outcomes and Pb are limited. However, Khalil et al. (2008) reported that elevated BLLs are associated with an increased risk of falls and nonspine fractures, further validating this concern. Moreover, osteoporotic patients have a decreased capacity for healing of fractures. Animal studies have also suggested that fracture healing is suppressed by Pb exposure (Carmouche et al. 2005). BMD measurements obtained from DXA is the current gold standard used to predict risk; however, we hypothesize that DXA predictions of fracture risk due to Pb exposure might underestimate actual fracture risk. The results of the present study suggest that Raman spectra of bone, if obtained transcutaneously, may be helpful in contributing to the prediction of fracture risk in patients exposed to Pb. Although these preliminary predictive results are compelling, they are based only on simple univariate regressions, and more complex multivariate prediction models could potentially provide more accurate predictions (Maher et al. 2011).

At the subtoxic Pb concentrations presented here, Pb affected bone homeostasis, resulting in decreased bone mass. Specifically, bone mass decreases were accompanied by a reduction in osteoblast number, suggesting a depression of bone formation, but we observed no significant change in osteoclast frequency or bone resorption. In addition, we found an increase in the number of adipocytes, suggesting a possible increase in adipogenesis. The increased expression of *PPAR*γ further supports this claim. Despite decades of knowledge of the detrimental effects of Pb on bone health, remarkably little is known of the mechanisms by which they occur. Because Wnt signaling is a very active anabolic pathway in bone, we hypothesized that that it is a target for Pb toxicity. To this end, we discovered a strong up-regulation of the Wnt inhibitory factor Scl and an analogous reduction of β-catenin. We also observed that Runx2 levels were depressed in Pb-treated rats. These findings support the hypothesis that Pb inhibits Wnt signaling, leading to reduced osteoblast activity. Moreover, our findings substantiate the *in vitro* acceleration of adipocyte formation and inhibition of bone nodule formation following Pb treatment. Depression of Wnt signaling has also been demonstrated to decrease adipogenesis by preventing the activation of proadipogenic factors that are required for progression of adipocyte formation (Li et al. 2007; Ross et al. 2000). This suggests that Pb inhibition of Wnt signaling can act as a molecular trigger to increase adipocyte frequency. Other environmental stressors, such as ethanol, have also been demonstrated to influence mesenchymal commitment via inhibition of Wnt signaling (Chen et al. 2010). Currently, there are human clinical trials using Scl and DKK antibodies to treat diseases of low bone mass signifying the pathogenic nature of these molecules (Fulciniti et al. 2009; Padhi et al. 2011). Thus, we believe that the observed depression of Wnt/β-catenin both *in vivo* and *in vitro* through Scl is an important mechanism for Pb toxicity in the skeleton. In all, our findings support the hypothesis that Pb can influence mesenchymal cell differentiation into osteoblasts or adipocytes through modulation of Wnt signaling.

The Pb-induced osteoporotic-like phenotype described here is remarkably similar and parallels symptoms and sequelae of human osteoporosis. Specifically, the human condition of osteoporosis is often characterized by a decreased number of bone-forming cells that causes depression of formation, unfilled resorptive surfaces, and a conversion of bone marrow to increased adiposity (Gimble et al. 2006; Nuttall and Gimble 2004; Syed et al. 2008; Verma et al. 2002). All of these observations were present in our Pb-treated rat population. These two converging pathologies, depressed bone formation and increased adipogenesis, helps further our understanding of how Pb is a risk factor for osteoporosis by unbalancing bone homeostasis by reducing of Wnt signaling. These findings indicate that Pb has a detrimental impact in bone at levels previously thought safe. This study, in conjunction with others, indicates that low-level Pb effects need to be considered when evaluating Pb policy concerning adverse effect levels.

## Correction

In the manuscript originally published online, J. Edward Puzas’s name was incorrect in the author list. It has been corrected here.

## Supplemental Material

(1.8 MB) PDF
