# Errata

**DOI:** 10.1289/ehp.121-a43

**Published:** 2013-02-01

Beier et al. [Environ Health Perspect 121:97–104 (2013)] have noted that the wrong control photomicrograph was used in their Figure 4A. The corrected figure is presented below.

The authors regret the error.

**Figure:**
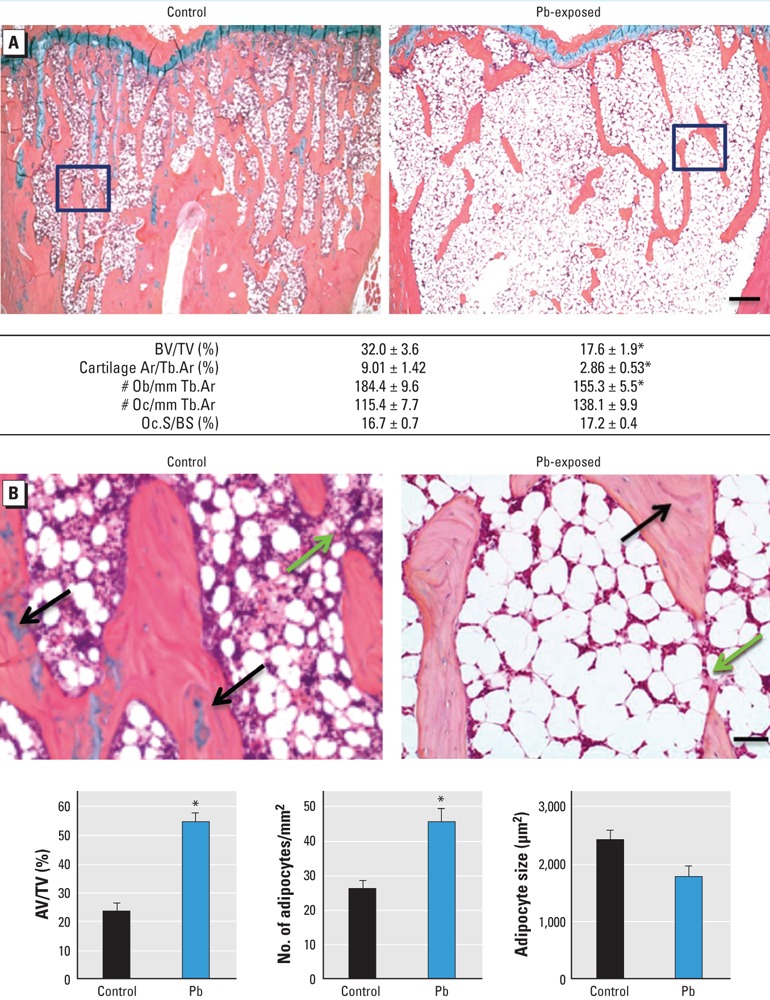
Changes in bone and adipogenic histomorphometric parameters in Pb-exposed rats compared with controls. (A) Sections of trabecular bone in the metaphyseal region of proximal tibia from control and Pb-treated rats (top) were evaluated for the following bone properties (bottom): BV/TV, cartilage area to trabecular area (Cartilage Ar/Tb.Ar), osteoblast number to trabecular area (Ob/Tb.Ar), osteoclast number to trabecular area (Oc/Tb.Ar), and osteoclast surface to bone surface (Oc.S/BS). (B) Images magnified from areas indicated by black boxes in (A) show fatty bone marrow changes in rat tibia (top). Green arrows highlight areas of unfilled tunneling and resorption space in Pb exposures, and black arrows indicate cartilage bars in trabecular bone. Bone sections were evaluated for adipocyte content (bottom). AV/TV, adipose volume to total volume. Data represent mean ± SE (n = 4 rats/group). Bar = 100 μm in (A) and 500 μm in (B). *p < 0.05.

The May 2012 Focus article “Offshore Exploration in the Arctic: Can Shell’s Oil-Spill Response Plans Keep Up?” [Environ Health Perspect 120:A194–A199 (2012)] contained a typographical error on A197. The figure “4.9 billion” should have read “4.9 million.”

EHP regrets the error.

