# Supplementary material for: Heavy Metal Lead Exposure, Osteoporotic-like Phenotype in an Animal Model, and Depression of Wnt Signaling
Source: Environ Health Perspect. 2012 Oct 19;121(1):97–104. doi: 10.1289/ehp.1205374 (PMC3552813; doi:10.1289/ehp.1205374)
Supplement: (1.8 MB) PDF [file ehp.1205374.s001.pdf]

Supplemental Material

Heavy Metal Lead Exposure, Osteoporotic-like Phenotype in an Animal Model, and Depression of Wnt Signaling

Authors:

Eric E Beier [1,2], Jason R Maher [3], Tzong-Jen Sheu [1], Deborah A Cory-Slechta [2], Andrew J Berger [3], Michael J Zuscik [1], Edward J Puzas [1,2]

Table of Contents

1. Title Page
2. Table of Contents
3. Raman spectroscopy analysis
4. References
5. Supplemental Table S1
6. Supplemental Table S2
7. Supplemental Table S3
8. Supplemental Figure S1

Supplemental details of Raman spectroscopy analysis: Raman spectroscopy is capable of determining the biochemical composition of bone and has been applied to study both normal and osteoporotic tissue (Akkus et al. 2004; Boivin and Meunier 2003; Carden and Morris 2000). Differences in mineral and protein content between the rat femurs were characterized by metrics related to bone biochemistry. The mineral-to-matrix ratio (MTMR; PO_4^{3-} / CH_2 peak area ratio) describes the degree of phosphate mineralization. The carbonate-to-phosphate ratio (CTPR; CO_3^{2-} / PO_4^{3-} peak area ratio) describes the amount of carbonate substitution in the hydroxyapatite crystal lattice. Collagen maturity (1660 cm^{-1} / 1690 cm^{-1} peak intensity ratio) describes the ratio of mature (pyridinoline) to immature (dehydro-dihydroxylysinonorleucine) collagen cross-links. Finally, crystallinity (inverse of the PO_4^{3-} peak width at half-max intensity) is a measurement of mineral maturity, crystallite size, and the amount of substitution into the apatitic lattice (Faibish et al. 2006; Paschalis et al. 2001). Metrics were normalized by the average value calculated among rats in the control group.

Supplemental References:

Akkus O, Adar F, Schaffler MB. 2004. Age-related changes in physicochemical properties of mineral crystals are related to impaired mechanical function of cortical bone. Bone 34(3):443-453.

Boivin G, Meunier PJ. 2003. The mineralization of bone tissue: a forgotten dimension in osteoporosis research. Osteoporos Int 14 Suppl 3:S19-24.

Carden A, Morris MD. 2000. Application of vibrational spectroscopy to the study of mineralized tissues (review). J Biomed Opt 5(3):259-268.

Faibish D, Ott SM, Boskey AL. 2006. Mineral changes in osteoporosis: A review. Clin Orthop Relat Res 446:28-38.

Paschalis EP, Verdelis K, Doty SB, Boskey AL, Mendelsohn, Yamauchi M. 2001. Spectroscopic characterization of collagen cross-links in bone. J Bone Miner Res 16(10):1821–1828.

Supplemental Table S1

Primer Sequences used for Real-Time Polymerase Chain Reaction analyses.

**Gene**	**Forward Primer**	**Reverse Primer**
Adipsin (cfd)	CGGATGACGACTCTGTGCAG	CATCGCTTGTAGGGTTCAGGG
Alkaline phosphatase	TCCTGACCAAAAACCTCAAAGG	TCGTTCATGCAGAGCCTGC
aP2	TGGGGACCTGGAAACTCGT	TCTCTGACCGGATGACGAC
β-actin	TGTTACCAACTGGGACGACA	CTGGGTCATCTTTTCCAGGT
β-catenin (mouse)	ATGGAGCCGGACAGAAAAGC	GAATCCAAGTAAGACTGCTGCT
β-catenin (rat)	GCTGACCTGATGGAGTTGGA	TCTTCTTCCTCAGGATTGCC
C/EBP α	ATAAGAACAGCAACGAGTACC	GCGGTCATTGTCACTGGTC
C/EBP δ	CCACGACCCCTGCCATGTAT	TGTGATTGCTGTTGAAGAGGTC
Osteocalcin	AGGGAGGATCAAGTCCCG	GAACAGACTCCGGCGCTA
Osterix	ACTGGCTAGGTGGTGGTCAG	GGTAGGGAGCTGGGTTAAGG
PPAR-γ	TATGGGTGAAACTCTGGGA	TGGCATCTCTGTGTCACCAT
Runx-2	GCCGGGAATGATGAGAACTA	GGACCGTCCACTGTCACTTT
Type 1 collagen	GCATGGCCAAGAAGACATCC	CCTCGGGTTTCCACGTCTC

Supplemental Table S2

Biomechanical strength of lumbar vertebrae and long bones are decreased in Pb-treated rats.

Compression[a]	Stiffness (N/mm)	Max Load (N)	Energy to Failure (mJ)	Yield Force (N)
Control	700.99 ± 61.91	280.69 ± 30.93	86.50 ± 10.53	158.00 ± 33.94
Pb-exposed	507.03 ± 20.12*	181.61 ± 21.05*	68.25 ± 6.07	123.43 ± 36.52
**4-point Bending[b]**				
Control	637.73 ± 36.91	264.42 ± 10.04	73.53 ± 7.39	242.99 ± 6.33
Pb-exposed	590.45 ± 79.40	204.58 ± 17.45**	50.52 ± 5.83*	178.73 ± 16.37**

Data represent mean ± SEM for 6 rats/group for compression and 9 rats/group for bending.

[a]Third lumbar vertebra were subjected to compression to failure testing. [b]Femurs were subjected to 4-point bend testing. *Significant at $p < 0.05$, **significant at $p < 0.005$.

Supplemental Table S3

Biomechanical strength of lumbar vertebrae and long bones are decreased in Pb-treated rats.

Raman Metric	Control	Pb-exposed
MTMR	1.00 ± 0.13	0.76 ± 0.09*
CTPR	1.00 ± 0.07	1.13 ± 0.08*
collagen maturity	1.00 ± 0.10	1.47 ± 0.19*
crystallinity	1.00 ± 0.002	0.992 ± 0.005*

Biochemical parameters of each group derived from the Raman spectra and normalized to control values. Data represent mean ± SEM for 4 rats/group, *$p < 0.05$

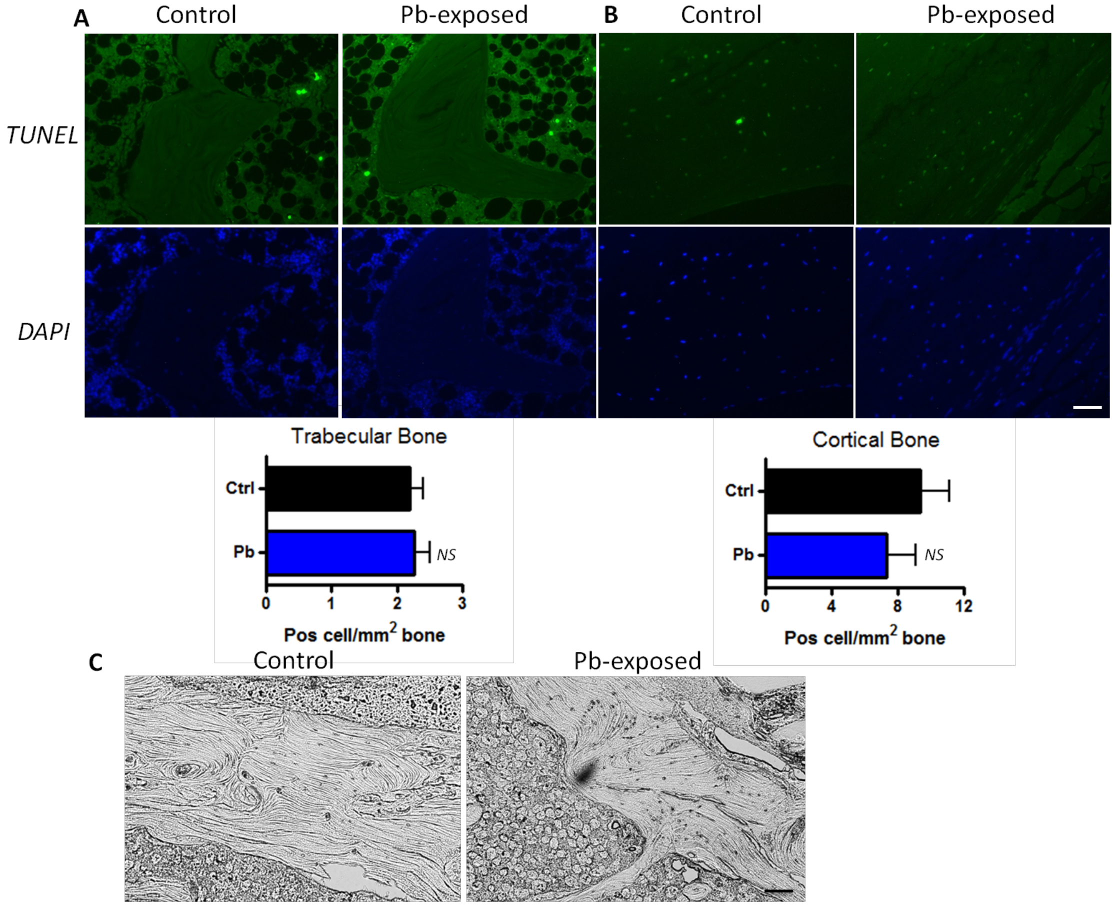

Supplemental Figure S1

Pb exposure had no effect on cell viability and bone structure. No change was seen in positive TUNEL staining between 0-Pb and 50-Pb treated rats in either trabecular (A) or cortical bone (B). Normal woven bone was observed in cortical bone (C). Data represent mean ± SEM for 3 samples. Scale bar: (A, B) 500 µm, (C) 100 µm, n=3.
